# Selection on Sperm Count, but Not on Sperm Morphology or Velocity, in a Wild Population of *Anolis* Lizards

**DOI:** 10.3390/cells10092369

**Published:** 2021-09-09

**Authors:** Ariel F. Kahrl, Matthew C. Kustra, Aaron M. Reedy, Rachana S. Bhave, Heidi A. Seears, Daniel A. Warner, Robert M. Cox

**Affiliations:** 1 Department of Zoology, Stockholm University, 106 91 Stockholm, Sweden; 2 Department of Biology, University of Virginia, Charlottesville, VA 22904, USA; mkustra@ucsc.edu (M.C.K.); aaronreedy@virginia.edu (A.M.R.); rsb7bz@virginia.edu (R.S.B.); heidiseears@gmail.com (H.A.S.); rmc3u@virginia.edu (R.M.C.); 3 Department of Ecology and Evolutionary Biology, University of California, Santa Cruz, CA 95064, USA; 4 Department of Biological Sciences, Auburn University, Auburn, AL 36849, USA; daw0036@auburn.edu

**Keywords:** sperm competition, multivariate selection, *Anolis*, ejaculate traits, sperm count, wild population

## Abstract

Sperm competition is a widespread phenomenon that shapes male reproductive success. Ejaculates present many potential targets for postcopulatory selection (e.g., sperm morphology, count, and velocity), which are often highly correlated and potentially subject to complex multivariate selection. Although multivariate selection on ejaculate traits has been observed in laboratory experiments, it is unclear whether selection is similarly complex in wild populations, where individuals mate frequently over longer periods of time. We measured univariate and multivariate selection on sperm morphology, sperm count, and sperm velocity in a wild population of brown anole lizards (*Anolis sagrei*). We conducted a mark-recapture study with genetic parentage assignment to estimate individual reproductive success. We found significant negative directional selection and negative quadratic selection on sperm count, but we did not detect directional or quadratic selection on any other sperm traits, nor did we detect correlational selection on any trait combinations. Our results may reflect pressure on males to produce many small ejaculates and mate frequently over a six-month reproductive season. This study is the first to measure multivariate selection on sperm traits in a wild population and provides an interesting contrast to experimental studies of external fertilizers, which have found complex multivariate selection on sperm phenotypes.

## 1. Introduction

The extreme diversity observed in sexually selected traits among species is often attributed to strong directional selection that drives trait exaggeration [1,2]. While sexually selected traits are often associated with mating success (i.e., precopulatory selection), there is growing appreciation that postcopulatory selection also leads to exaggeration of traits, particularly those associated with male ejaculates [2,3]. Postcopulatory selection occurs after mating via sperm competition and cryptic female choice, meaning that sperm may simultaneously experience (1) selection to outcompete rival sperm, and (2) selection arising from females biasing fertilization [4,5,6]. The opportunity for postcopulatory sexual selection is high in promiscuous species, where males and females each mate with multiple partners [7,8]. Indeed, species with high incidence of multiple mating have larger testes [9,10,11,12,13], more efficient sperm-producing tissues [14,15,16], longer sperm [17], and faster sperm [18], relative to species with low incidence of multiple mating. However, ejaculates are complex and present many potential targets for selection, and the ways in which postcopulatory selection acts on the individual components of an ejaculate can be highly variable across species [18]. Thus, estimates of multivariate selection on ejaculate traits are needed to fully understand how these traits evolve [17,19].

Ideally, males would invest in ejaculate quantity and quality to maximize fitness. However, functional constraints and energetic trade-offs among ejaculate traits and other traits that are important for fitness can limit maximal investment into different ejaculate components [20]. The rich theoretical literature exploring how males should optimally allocate reproductive resources to ejaculate traits (reviewed in [20]) posits that the risk of sperm competition (the likelihood that a female will remate with a second male) and the intensity of sperm competition (the number of males competing for fertilization with a single female) influence how much energy males should optimally invest in a given ejaculate, be it in sperm size or sperm number [13,21,22,23,24,25,26]. This dynamic is especially true for species in which males mate with multiple females and cannot effectively monopolize access to females. In this case, as the intensity of sperm competition increases, optimal investment per ejaculate is actually expected to decrease because males should retain ejaculate resources for multiple matings [26]. Within ejaculates, whether sperm number and/or sperm size are predicted to increase with the risk of sperm competition is partly attributed to whether males compete under a raffle model of competition (where sperm do not compete for storage and fertilization is proportional to sperm number), or a displacement model of competition (where sperm displace one another from sperm storage sites) [27,28]. Increasing sperm size may often be beneficial in species where sperm displacement is likely, such as invertebrates with small sperm storage sites [27,28]. By contrast, increasing sperm number is often more important in external fertilizers and larger vertebrates, where dilution of an ejaculate can occur in the environment or the female reproductive tract [27,28]. However, a recent meta-analysis indicates that sperm total length, as well as the lengths of sperm components, generally increase with level of sperm competition, even in larger vertebrates [17]. 

Because sperm traits are often phenotypically correlated with one another, direct selection on some traits may produce indirect selection on others [29,30,31,32,33,34]. Moreover, male fitness may actually depend on combinations of sperm traits [35,36], resulting in a complex selective landscape with fitness optima that can only be detected in a multivariate framework [37]. Because of this complexity, studies have begun to measure multivariate selection on ejaculate traits to partition direct and indirect selection and quantify correlational selection acting on combinations of ejaculate traits [29,30,31,32,33,34,37]. However, all current multivariate selection analyses have been conducted in captivity, most have involved external fertilizers, and the majority have been conducted in an experimental setting in which the number of competing ejaculates, the number of sperm in each ejaculate, and/or the number of matings are fixed. If fitness measurements are derived from an artificially low number of mating events, this may skew our view of selective optima towards males that invest more in a single ejaculate (whether that be in quantity or quality of sperm), rather than males who invest less in each ejaculate but produce many more ejaculates. Selection in the wild may operate very differently, but few data are available to test this possibility. 

When selection is measured over a longer reproductive interval in which males can potentially mate frequently and with many females, the strength and direction of selection, as well as the sperm traits targeted by selection, may differ from what is observed in captivity. Theory suggests that, in wild populations with low female monopolization and high mating rate [26], selection should favor males that do not invest too much in a single ejaculate, which would allow them to increase their mating frequency and the number of ejaculates produced [38,39,40]. If increased mating frequency improves male reproductive success, selection may favor a reduction in sperm count in order to increase the number of mating opportunities. Indeed, in Soay sheep (*Orvis aries*), dominant males who are able to mate frequently sire the most offspring, but suffer from sperm depletion as the mating season progresses [41]. This suggests that sperm production may be an important target of selection in wild populations. Although correlations between sperm traits and reproductive success have been examined in some wild populations (e.g., [42,43,44]), most have estimated selection on a single sperm trait (sperm velocity, morphology, or count), and may have confounded direct and indirect selection, or missed correlational selection on trait combinations. Therefore, a multivariate selection analysis on ejaculate traits in a wild population is crucial for improving our understanding of how selection acts on ejaculate traits. 

In this study, we used genetic quantification of reproductive success to measure selection on a suite of ejaculate traits in a wild island population of brown anole lizards (*Anolis sagrei*). Brown anoles likely experience strong postcopulatory selection because they are highly promiscuous [45,46], can store sperm for several months [45,47,48], live in dense populations [49], and mate frequently during their extended reproductive season [50,51]. In an experimental laboratory setting, several sperm traits (i.e., sperm head length, midpiece length, and sperm count) were associated with reproductive success when two competing males were each allowed to mate once with a female [47]. In addition, a comparative study across *Anolis* revealed that testis size evolves over three times faster than sperm morphology in this genus [52], suggesting that sperm production may be the primary target of postcopulatory selection. Based on this prior experimental and comparative work, we predicted that sperm count would be under selection in wild brown anoles. On one hand, promiscuous mating systems and high incidence of multiple paternity may favor an increased number of sperm per ejaculate, resulting in positive directional selection on sperm count. Alternately, frequent mating over a lengthy reproductive season may instead favor an increased number of ejaculates over a high number of sperm per ejaculate, resulting in negative directional selection on sperm count. If these selective forces are both at play, or if there is an intermediate level of sperm production that is optimal, we predicted that we would see negative quadratic (i.e., stabilizing) selection on sperm count. Given that other sperm traits (i.e., sperm head length, midpiece length, and sperm count) have previously been associated with proportional paternity in controlled mating experiments on this species [47], we also tested whether these traits were associated with reproductive success in the wild. To compare patterns of selection on sperm traits from a wild population to previous laboratory tests, we also asked whether selection acts predominantly on individual ejaculate traits or simultaneously on several traits and/or trait combinations by examining selection in both univariate and multivariate frameworks. 

## 2. Materials and Methods

### 2.1. Collection of Individuals and Sperm Samples

As part of an ongoing mark-recapture project, we made four collection trips in 2015 (April, May, July-August, and October), collecting nearly all adult male (*n* = 331) and female (*n* = 638) *A. sagrei* from a small (1720 m^2^) island population within the Guana Tolomato Matanzas National Estuarine Research Reserve (Palm Coast, Florida, 29°63′ N, 81°21′ W). During two of these trips (July–August and October 2015) and an additional two trips the following year (March–April and May 2016), we captured 2060 juveniles that hatched in 2015 and recaptured all surviving adult males and females. All adults present in 2015 and all juveniles hatched in 2015 were used for genotyping and parentage analysis (see below). We collected a small tissue sample (5–10 mm tail tip) from each individual and preserved it in ethanol at −20 °C until DNA extraction. During our focal census in May (19–28 May 2015) we captured *n* = 211 adult males and *n* = 465 females. We measured snout-vent length (SVL, nearest mm) and body mass (nearest 0.01 g) for each lizard, then kept males in isolation overnight prior to collection of a sperm sample, which we obtained by depressing the abdomen and collecting the ejaculate into a microcapillary tube [47].

### 2.2. Measuring Sperm Traits

In our May 2015 census, we collected ejaculate samples from the majority of the 211 males that we captured (*n* = 202, 95%). We measured sperm velocity and linear movement by suspending ejaculates in 1000 μL of Dulbecco modified eagle medium (Gibco, ThermoFisher Scientific, Waltham, MA, USA) and immediately adding 50 μL of this suspension to a covered well slide. We recorded a 1-min video of each sample at 25 frames per second and 40× magnification using an AmScope digital camera (AmScope, Irvine, CA, USA) with the software ToupView (ToupTek Photonoics, Zhejiang, P.R. China). We then tracked 15 cells per individual for at least 1.8 s (minimum = 45 frames, mean = 54.8 frames) using the Manual Tracking plugin in ImageJ (NIH, Bethesda, MD, USA). We selected cells by starting in the upper left quadrant of the first frame of the video and tracking every motile cell in that area. We then moved clockwise through each quadrant of the video frame until we had measured the tracks of 15 cells, excluding any that were immobile, visibly impeded by another cell, or stuck to the slide/coverslip. We excluded *n* = 17 males because their sperm were too crowded on the slide or were non-motile, preventing accurate measurements of velocity. We used the ImageJ plugin AG paNoel v1.0.0 (Universidad Nacional de Cordoba, Argentina; https://www.iibyt.conicet.unc.edu.ar/software/, accessed on: 10 August 2021) to calculate two different aspects of sperm movement, the average path velocity (VAP, µm/sec) and linearity (the percent forward progression of the cell) of each cell using the cartesian coordinates of the tracks. This software allows for VAP to be accurately calculated from videos with lower framerates (5–30 fps) using a modification called VAPi, which adds an additional smoothing step. We calculated an average VAPi and average linearity from 15 cells for each male. 

From the same sperm sample used for velocity and linearity, we fixed the remaining cells from each male in 4% paraformaldehyde. To measure sperm count we pipetted 10 μL onto a hemocytometer counted the number on the slide, and then calculated the number of sperm in the entire sample. We then dried the remaining sample onto slides to measure sperm morphology (*n* = 198 males). We have previously shown that sperm counts estimated from these ejaculates are correlated with the amount of sperm transferred during mating in the lab (*r*^2^ = 0.49, *p* = 0.001 [47]), however this has not been confirmed for our wild population. Four males had little to no sperm on the slide and we were therefore unable to measure their sperm morphology. We stained these slides with Sperm BlueTM (Microptic SL, Barcelona, Spain) and imaged 15 cells per male at 100× magnification with an Olympus Magnafire camera (Olympus America, Melville, NY, USA) using differential interference contrast microscopy. To quantify sperm morphology, we measured the length of the sperm head (which contains the acrosome and nucleus), midpiece (which contains the beginning of the axoneme and the cell’s mitochondria), and flagellum (which is the remainder of the axoneme, containing both the principal piece and endpiece) of 15 cells per male using ImageJ (NIH, Bethesda, MD, USA), then calculated the mean length of the sperm head, midpiece, and flagellum for each male and used these values in our subsequent analyses (Supplementary Materials, Table S1) [47,53]. Finally, although other studies have demonstrated that unbiased measurements of sperm velocity can be estimated using a low number of cells [54], we used a resampling procedure to confirm that 15 cells were sufficient to approach asymptotically low levels of variance in our estimates of sperm morphology, velocity (VAPi), and linearity. We did this by resampling, with replacement, parameter values at increasing sample sizes (2–15) for each male, with 10,000 simulations at each sample size. We then calculated the average standard deviation of each increment across all males (Supplementary Materials Figure S1).

### 2.3. Genotyping and Parentage Assignment

We extracted DNA from ethanol-preserved *A. sagrei* tail tissue samples by adding up to 3 mg of tissue into 150 μL of 5–10% by weight Chelex 100 resin (BIO-RAD, Hercules, CA, USA) with 1.4 μL of 10 mg/mL Proteinase-K (VWR, Solon, OH, USA). We incubated this mixture for 240 min at 55 °C, followed by 10 min at 99 °C. Nucleic acid purification was carried out using 1.8 × volume of AMPure XP beads (Beckman Coulter, Brea, CA, USA), with samples eluted in 20 μL 1× TE (Fisher BioReagents, Fair Lawns, NJ, USA) buffer to raise the DNA concentration to at least 10 ng/μL. We genotyped samples using the Genotyping-in-Thousands by sequencing (GT-seq) method [55], utilizing a custom panel of 215 SNP markers that we developed from RAD-Seq data (H. Seears, unpublished). Briefly, we used a multiplex PCR to amplify all 215 loci simultaneously, and a second PCR to attach well-specific and plate-specific indices to each sample (see [55] for details). We standardized DNA concentrations of PCR products across samples using SequalPrep Normalization Kit (ThermoFisher Scientific, Waltham, MA, USA). These libraries were then pooled and cleaned using 1.8 x volume of AMPure XP beads (Beckman Coulter, Brea, CA, USA). Sequencing was performed on an Illumina HiSeq X with 2 × 150 bp paired-end reads. We checked the raw Illumina reads for quality using the software FastQC (https://www.bioinformatics.babraham.ac.uk/projects/fastqc/, accessed on: 5 March 2018) [56], and performed genotyping using two scripts from the GT-seq pipeline; GTseq_Genotyper_v3.pl and GTseq_GenoCompile_v3.pl [55]. Any individuals found to have >50% missing loci were excluded from subsequent analyses. 

Using these SNP genotypes, we performed parentage assignment with the software SNPPIT [57], which implements a likelihood-based method to infer parentage in trios (both parents and one offspring). We used an estimated genotyping error rate of 1% (i.e., a per-allele rate of 0.5%), and an additional exclusion threshold of 50 missing loci (23% of loci) for adults (but not offspring), above which an adult would not be considered as a putative parent. Adult males that were excluded from parentage assignment due to missing loci were excluded from the selection analysis as well (*n* = 10). SNPPIT summarizes confidence in parentage assignments in terms of the false discovery rate (FDR), and we accepted parentage calls with an FDR < 0.005. Based on the parent-offspring pairs, we calculated the number of offspring and number of unique mates per individual. Any adults that were successfully sequenced and included in the SNPPIT analysis but were not assigned offspring were considered to have 0 offspring and 0 mates. 

### 2.4. Estimating Linear and Non-Linear Selection

We conducted both univariate and multivariate selection analyses for a total of 171 males on a suite of sperm traits in R version 3.5.2 [58]. Details on the individuals excluded from the study because of missing data are available in Supplementary Materials, Table S2. We estimated univariate linear (*s*) and non-linear (*c*) selection differentials, and multivariate linear (*β*) and non-linear (*γ*) selection gradients as outlined by Lande and Arnold [59]. We first standardized each trait (sperm count, sperm velocity, sperm linearity, sperm head length, midpiece length, and flagellum length) to a mean of 0 with a standard deviation of 1, and calculated relative fitness for each male as the number of offspring he sired in 2015 divided by the mean number of offspring across all males [59]. We then used ordinary least-squares regressions of relative fitness on individual traits to estimate univariate selection differentials, and a multiple regression including all traits to estimate multivariate selection gradients. We estimated *s* and *β* from models that contained only linear terms, and *c* and *γ* from models that included both linear and quadratic terms. Regression coefficients and their associated standard errors were doubled to obtain estimates of quadratic selection [60]. The multivariate model used to estimate quadratic selection gradients (*γ*_ii_) also included cross-product terms to estimate correlational selection (*γ*_ij_) on trait combinations. Because relative fitness was highly skewed, we used generalized linear models with quasi-Poisson error distributions and log links to assess the significance of the selection estimates. Because ejaculate traits are often correlated with each other, we assessed the level of multicollinearity among our traits by calculating variance inflation factors for both linear (all VIF < 1.4) and non-linear models (all VIF < 2.8). To aid in the interpretation of our selection analyses, we explored the potential relationships between our sperm traits by calculating partial correlation coefficients from a multivariate correlation model that included all sperm traits. Coefficients were generated in R with the *stats* package [58].

We used a nonparametric analysis to visualize the fitness surface for sperm count, the only trait that was under significant selection [61,62]. Specifically, we used a univariate generalized additive model (GAM) to estimate the cubic spline of relative fitness as a function of sperm count, implemented in the R package *mgcv* [63]. We selected the smoothing parameter, K, that minimized the generalized cross-validation (GCV) score (Supplementary Materials, Figure S2).

## 3. Results

### 3.1. Parentage

We obtained genotype data for 2868 of the 3029 *A. sagrei* individuals collected in the field between April 2015 and May 2016 (90.4% success rate). We were unable to genotype 77 individuals (19 male, 32 female, 26 juveniles) due to insufficient DNA, and we excluded an additional 84 individuals after genotyping (5 male, 26 female, 53 juveniles) due to missing loci (Supplementary Materials, Table S2). Parentage assignment using SNPPIT [57] was carried out with 887 adults as putative parents (307 male, 580 female) and 1981 offspring. This skew in the sex ratio among adults is primarily due to differential survival between males and females [64]. Some of these adult males (*n* = 96) are not included in the selection analysis because they were not captured and phenotyped in May of 2015 but were collected in other censuses in 2015 and included for parentage assignment. Successful parentage assignments were made for 1185 offspring at an FDR < 0.005. The remaining 796 offspring had no parental matches. Of the adult population, 581 individuals were identified as parents (200 sires, 381 dams), 19 individuals (5 males, 14 female) were excluded due to missing data at >50 loci and 287 adults (102 male, 185 female) were not matched to any offspring (Supplementary Materials, Table S2). Many offspring may not have been assigned to parents because SNPPIT requires that both parents are present in the dataset to successfully assign parentage, and 29 males and 72 females (see above) were not included as potential parents. For individuals who were successfully genotyped, males had a mean of 3.85 mates (range: 0–16) and females had a mean of 1.55 mates (range: 0–8). For the subset of adults that produced at least two progeny (i.e., for the subset from which it was possible to detect multiple mates), males had a mean of 4.55 mates (range: 1–16) whereas females had a mean of 2.45 mates (range: 1–8).

### 3.2. Estimating Linear and Non-Linear Selection

**Figure 1:**
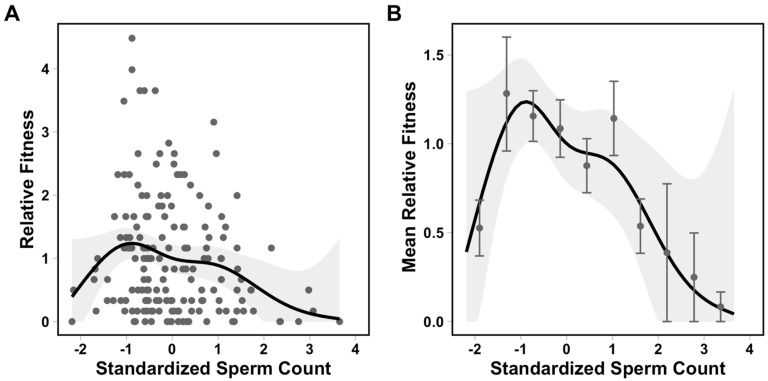
Univariate fitness surface for sperm count, which was characterized by both negative linear selection and negative quadratic selection. In both plots, the solid line represents the predicted relative fitness from the GAM model with the best fit (K = 6; Supplementary Materials Figure S1), and the grey shaded area represents the 95% confidence interval of the model prediction. Panel (**A**) shows each individual male as a data point, while panel (**B**) summarizes males into 10 bins of approximately equal size, with mean ± SE for each group to better visualize the fitness surface.

We found significant negative directional selection on sperm count (univariate: *s* = −0.179 ± 0.070, *F*_1,169_ = 6.489, *p* = 0.011; multivariate: *β* = −0.168 ± 0.0764, *F*_1,164_ = 5.269, *p* = 0.021), but linear selection gradients were not significant for any other sperm trait (Table 1). We also found significant negative quadratic selection on sperm count (univariate: *c* = −0.219 ± 0.080, *F*_1,169_ = 7.399, *p* = 0.001; multivariate *γ* = −0.162 ± 0.104, *F*_1,157_ = 6.058, *p* = 0.014), which indicates that selection reduced variance in sperm count (Figure 1, Table 1). We did not find any other significant quadratic selection gradients for other sperm traits, nor did we detect significant correlational selection on trait combinations (Table 1). To ensure that selection on sperm count was not being driven by underlying effects of body size or age (which is roughly approximated by body size), we included snout-vent length (SVL) in additional multivariate analyses of linear and quadratic selection on sperm count. We found no significant selection on SVL, and we obtained very similar estimates (in both magnitude and direction) of selection on sperm count (Table 2).

We detected significant phenotypic correlations between several sperm traits, especially between sperm head, midpiece, and tail length, and between sperm velocity and linearity (Table 3). We also found a significant positive correlation between sperm count and sperm velocity, a significant negative correlation between sperm count and midpiece length, and a significant positive correlation between sperm head length and sperm velocity (Table 3). However, these significant correlations were generally modest in magnitude (0.173 < *r* < 0.287; with the exception of velocity and linearity, *r* = 0.638), and VIF scores revealed no evidence of multicollinearity among these traits (Table 3). Moreover, univariate and multivariate estimates of selection were similar for most traits (Table 1), indicating that phenotypic correlations did not generally result in appreciable indirect selection.

## 4. Discussion

Our study is the first to measure multivariate selection on a suite of ejaculate traits in a wild population. We found evidence for negative directional and negative quadratic selection on sperm count (Figure 1, Table 1) such that males with sperm counts slightly lower than average had the highest reproductive success in this population. Surprisingly, we did not detect significant linear or nonlinear selection on any other ejaculate trait, nor did we find evidence of correlational selection acting on trait combinations. Our results stand in contrast to many studies that have demonstrated complex multivariate selection on ejaculates via controlled, competitive-mating experiments in a semi-natural laboratory or experimental setting [29,30,31,32,33,34,37]. However, many of these laboratory studies examined external fertilizers and experimentally equalized the sperm counts of competing ejaculates prior to fertilization trials. By equalizing sperm count and competing ejaculates in a single event, these studies may have revealed more complex and subtle underlying patterns of selection on other ejaculate traits that might otherwise be masked by selection on sperm count. Sperm count is likely one of the primary targets of postcopulatory sexual selection, as testis size is frequently associated with the strength of postcopulatory selection [17,18,20,38]. Therefore, it may not be surprising that selection acts strongly on this trait in a wild population where individuals mate frequently over a lengthy reproductive season. 

Though sperm competition is predicted to favor an increase in sperm production, this increase may be due to selection for (1) few ejaculates with high sperm count, which may allow males to compete numerically in sperm competition [7,65], or (2) many ejaculates with lower sperm count, which may be favored when males mate frequently [38,39,40]. In comparison to other species of squamates, brown anoles have large testes for their body size [66], suggesting that they may have the capacity for high sperm production [67]. Brown anoles also have high mating rates [45,46] (0.11–0.18/per hour in natural setting [50]). Our parentage analysis revealed that those females who had at least two offspring had 2.45 mates on average, with >40% of these females mating with three or more males (range 1–8 mates). These are minimum estimates of multiple mating, as parentage alone will underestimate the actual number of mates for each female. Studies on this same population have detected that 24% and 47% of females mated within two-day intervals in May and July, respectively, with approximately 5% of females mating with at least two males over this same short interval (R.S. Bhave, unpublished). In our study, we found that males with lower sperm counts sired more offspring. One explanation for this result is that the males with the highest reproductive success had lower sperm counts simply because they have the highest mating rates in the population, and their sperm supply tended to be depleted in their reproductive tracts when sampling occurred. This pattern of sperm depletion has been observed in other species [41,68,69,70]. Alternatively, selection may have favored lower sperm counts for some combination of the reasons we discuss below.

In species where multiple mating occurs frequently, males experience high risk and intensity of sperm competition and must balance investment in their reproductive traits. As sperm competition intensity increases, the fitness benefit of producing ejaculates with high sperm counts plateaus and then declines [13,20,26]. Indeed, maximal sperm allocation per ejaculate is expected in situations with high risk and low intensity (didactic competition), and sperm allocation should drop as intensity increases (>3 males compete for fertilization) [23,25], which has been demonstrated in some species [71,72], but not others [73]. The rates of multiple mating we found in our population suggest that males in this population experience a high risk of sperm competition, and many of them will likely also face a high intensity of sperm competition. In this population, we previously found that males in high-density areas have lower sperm counts [74], potentially indicating that males allocate sperm based on their social environment [20,73,75]. If males are able to detect the risk and intensity of competition in their environment, they may maximize their fitness by mating frequently, but investing an intermediate amount of sperm per ejaculate to avoid sperm depletion [38,39,40]. Because we do not know how quickly males are able to replenish their sperm stores after mating, nor do we have detailed information about the mating histories of individual males, it is impossible to disentangle whether high-fitness males with below-average sperm counts are allocating strategically or experiencing sperm depletion due to frequent mating. Our analyses are also based on a single quantification of ejaculate traits at one point in the breeding season, and it is unclear whether and how these phenotypes might change over time. 

Of course, male fitness does not entirely depend on ejaculate production [64,76]. Before mating, males must attract mates and combat rival males [1,77], and after mating, males can increase their chances of siring offspring and lower the risk of sperm competition by mate guarding. The energy allocated for traits and behaviors involved in precopulatory selection and mate guarding may trade-off with the energetic demands of sperm production [21], resulting in lower sperm counts. However, if males are able to increase their mating opportunities and fitness by allocating more resources to precopulatory traits and behaviors relative to sperm production, males with a low to intermediate sperm count may have the highest fitness. Indeed, this pattern has been found in several species [41,78,79,80] where males invest in mate guarding or male–male competition, but not in sperm production, in environments with a high risk of sperm competition. Brown anoles are territorial, with high site fidelity in both sexes [74]. Additionally, female territories frequently overlap with several male territories [46,74]. Although direct mate guarding has not been reported in this species, males often engage in combat and defend areas in which females reside, frequently injuring other males [81,82]. If males pay a high cost defending territories and mates, this may trade-off with their ability to produce higher sperm counts and/or high-quality ejaculates.

Despite previous evidence of a link between fertilization success and sperm morphology in dyadic competitive trials using captive brown anoles [47], we found no evidence of selection on sperm morphology or velocity in the wild. In our previous study, male competitors were each only allowed to mate once with a female, and the female was allowed to lay eggs uninterrupted for up to five months. Similarly, studies of wild bird populations have found that sperm count is an important predictor of fertilization success [42], whereas laboratory and comparative studies have found different targets of selection (e.g., sperm velocity and morphology) [44,83,84]. When an artificially low number of ejaculates compete, there are fewer sperm competing overall, and sperm quality may confer higher reproductive success. For example, if the morphological components of sperm (in this case, midpiece and head size) play a role in sperm longevity [85,86], or sperm velocity [83,87,88], then we might detect an association between sperm morphology and reproductive success when sperm numbers are limited. However, when individuals in a wild population are able to mate multiple times over the entire reproductive season, the number of sperm competing in a female’s reproductive tract may become more important than the morphology, velocity, or quality of those sperm cells. Males that mate frequently with the same or multiple females may have a competitive advantage, supplying fresh sperm to the female and potentially displacing other sperm from storage [89,90]. Though we did not detect significant selection on sperm morphology or velocity in this study, it could be that our measurements of sperm velocity and morphology were not sensitive enough to detect a relationship with fitness. We also only quantified sperm phenotypes during one time point and it is possible that sperm traits and/or selection on sperm traits may change over the course of the breeding season. Additionally, our measurement of sperm velocity in vitro may not accurately reflect performance in the fluid and complex microenvironment of the female reproductive tract.

We have shown that sperm count experiences both negative linear and negative quadratic selection in a wild population of brown anoles, such that males with a low to intermediate sperm count have the highest reproductive success over the course of the season. We suggest that this pattern is linked with the high rates of mating observed in this species and the need to produce many ejaculates over a lengthy reproductive season. However, future work is needed to disentangle the effects of sperm count and mating rate and thereby understand the selective pressures on ejaculate traits and mating behaviors [20,39]. We hope that this becomes the first of many measurements of selection in the wild which can give context to correlations between ejaculate traits and the evolution of ejaculate traits among species.

## Figures and Tables

**Table 1:** Linear and non-linear selection differentials and gradients (±SEM) from univariate and multivariate selection analyses, respectively. Estimates were obtained using ordinary least-squares regressions with relative fitness (number of offspring sired relative to population mean) as the dependent variable and standardized male sperm traits and their quadratic terms as the independent variables (separate models were fit for linear and non-linear selection estimates). For estimates of γ, quadratic selection gradients are shown on the diagonal and represent disruptive (+) or stabilizing (−) selection on individual traits. Correlational selection gradients are shown above the diagonal and represent selection favoring positive or negative correlations between trait pairs. Significance of selection gradients was determined using generalized linear models with a quasi-Poisson distribution and a log link. Estimates with *p* < 0.05 are bolded.

	***s***	***β***	***c***			
**Traits**	**Linear** **(Univariate)**	**Linear** **(Multivariate)**	**Non-Linear** **(Univariate)**			
Sperm count	**−0.179 (±0.07)**	**−0.167 (±0.07)**	**−0.219 (±0.08)**			
Velocity (VAPi)	−0.021 (±0.07)	0.033 (±0.09)	−0.054 (±0.08)			
Linearity	−0.005 (±0.07)	0.009 (±0.09)	−0.012 (±0.08)			
Head length	−0.106 (±0.07)	−0.072 (±0.08)	−0.032 (±0.11)			
Midpiece length	0.106 (±0.07)	0.038 (±0.08)	−0.088 (±0.09)			
Flagellum length	−0.069 (±0.07)	−0.047 (±0.07)	0.024 (±0.09)			
	***γ***					
**Traits**	**Sperm Count** **(Multivariate)**	**Velocity (VAPi)** **(Multivariate)**	**Linearity** **(Multivariate)**	**Head Length** **(Multivariate)**	**Midpiece Length** **(Multivariate)**	**Flagellum Length** **(Multivariate)**
Sperm count	**−0.162 (±0.10)**	−0.027 (±0.13)	−0.084 (±0.11)	−0.008 (±0.07)	−0.037 (±0.08)	0.060 (±0.09)
Velocity (VAPi)		0.078 (±0.20)	−0.080 (±0.17)	−0.040 (±0.11)	0.188 (±0.13)	0.091 (±0.11)
Linearity			−0.034 (±0.17)	0.151 (±0.11)	−0.181 (±0.13)	0.037 (±0.11)
Head length				−0.058 (±0.12)	−0.040 (±0.08)	0.053 (±0.09)
Midpiece length					−0.168 (±0.12)	−0.055 (±0.09)
Flagellum length						−0.131 (±0.11)

**Table 2:** Linear and non-linear selection differentials and gradients (± SEM) from univariate and multivariate selection analyses on body size (SVL) and sperm count. Estimates were obtained using ordinary least-squares regressions with relative fitness (number of offspring sired relative to population mean) as the dependent variable, and standardized male sperm traits and their quadratic terms as the independent variables (separate models were fit for linear and non-linear selection estimates). Significance of selection gradients was determined using generalized linear models with a quasi-Poisson distribution and a log link. Estimates with *p* < 0.05 are bolded.

	*s*	*β*	*c*	*γ*	
Traits	Linear (Univariate)	Linear (Multivariate)	Non-Linear (Univariate)	Sperm Count (Multivariate)	SVL (Multivariate)
Sperm count	**−0.179 (±0.07)**	**−0.168 (±0.07)**	**−0.220 (±0.08)**	**−0.225 (±0.08)**	−0.022 (±0.07)
SVL	0.089 (±0.07)	0.056 (±0.07)	−0.048 (±0.08)		−0.040 (±0.08)

**Table 3:** Phenotypic correlations and trait collinearity. Variance inflation factors (VIF) are listed from the generalized linear model used to test significance of multivariate selection estimates. Phenotypic correlation coefficients (*r*) are listed for all pairwise combinations of ejaculate traits, with *p*-values in parentheses.

Trait	VIF	Velocity (VAPi)	Linearity	Head Length	Midpiece Length	Flagellum Length
Count	1.170	0.241 (0.001)	0.041 (0.593)	0.120 (0.117)	−0.249 (0.001)	0.039 (0.609)
Velocity (VAPi)	1.791		0.638 (0.001)	0.123 (0.107)	−0.130 (0.088)	0.135 (0.076)
Linearity	1.690			0.121 (0.113)	−0.118 (0.122)	0.101 (0.185)
Head length	1.136				−0.287 (0.001)	0.174 (0.023)
Midpiece length	1.223					−0.231 (0.003)
Flagellum length	1.104					

## Data Availability

Data are available on the Open Science Framework: https://osf.io/c4wn5/, accessed on: 4 September 2021.

## Supplementary Materials

The following are available online at https://www.mdpi.com/article/10.3390/cells10092369/s1. Figure S1. Repeated resampling of sperm morphology, velocity, and linearity. Figure S2. Generalized cross validation scores for smoothing parameter. Table S1. Summary statistics of sperm parameters and paternity. Table S2. Sample sizes for genotyping and phenotyping with exclusion criteria.
