# Supplementary material for: Selection on Sperm Count, but Not on Sperm Morphology or Velocity, in a Wild Population of Anolis Lizards
Source: Cells. 2021 Sep 9;10(9):2369. doi: 10.3390/cells10092369 (PMC8464841; doi:10.3390/cells10092369)
Supplement: Supplementary file 1 [file cells-10-02369-s001.zip › cells-1274588-supplementary.pdf]

cells

Article

Selection on sperm count, but not on sperm morphology or velocity in a wild population of *Anolis* lizards

Ariel F. Kahrl [1,2*], Matthew C. Kustra [2,3], Aaron M. Reedy [2,4], Rachana S. Bhave [2], Heidi A. Seears [2], Daniel A. Warner [4], and Robert M. Cox[2]

[1] Stockholm University, Zoologiska institutionen: Etologi, Stockholm, 106 91, Sweden
[2] Department of Biology, University of Virginia, Charlottesville, VA 22904, USA
[3] Department of Ecology and Evolutionary Biology, University of California, Santa Cruz, CA 95064, USA
[4] Department of Biological Sciences, Auburn University, Auburn, AL 36849, USA
* Correspondence: ariel.kahrl@zoologi.su.se

Supplemental Figures and Tables

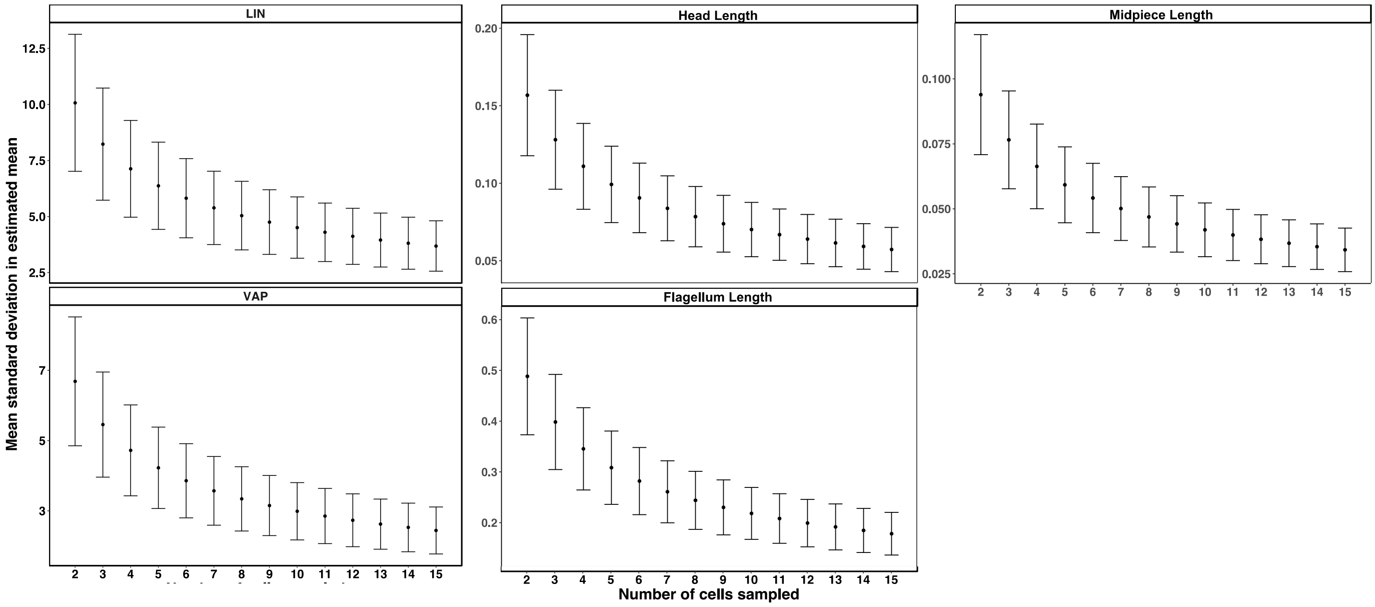

Figure S1. We used repeated resampling to validate the use of 15 sperm cells for our measurement of linearity (LIN) and sperm velocity (VAPi) and sperm head, midpiece and flagellum lengths. For each increment and male, we resampled 10,000 iterations and then calculated the standard deviation of the estimated means. Points are the mean standard deviation across males and error bars are one standard deviation.

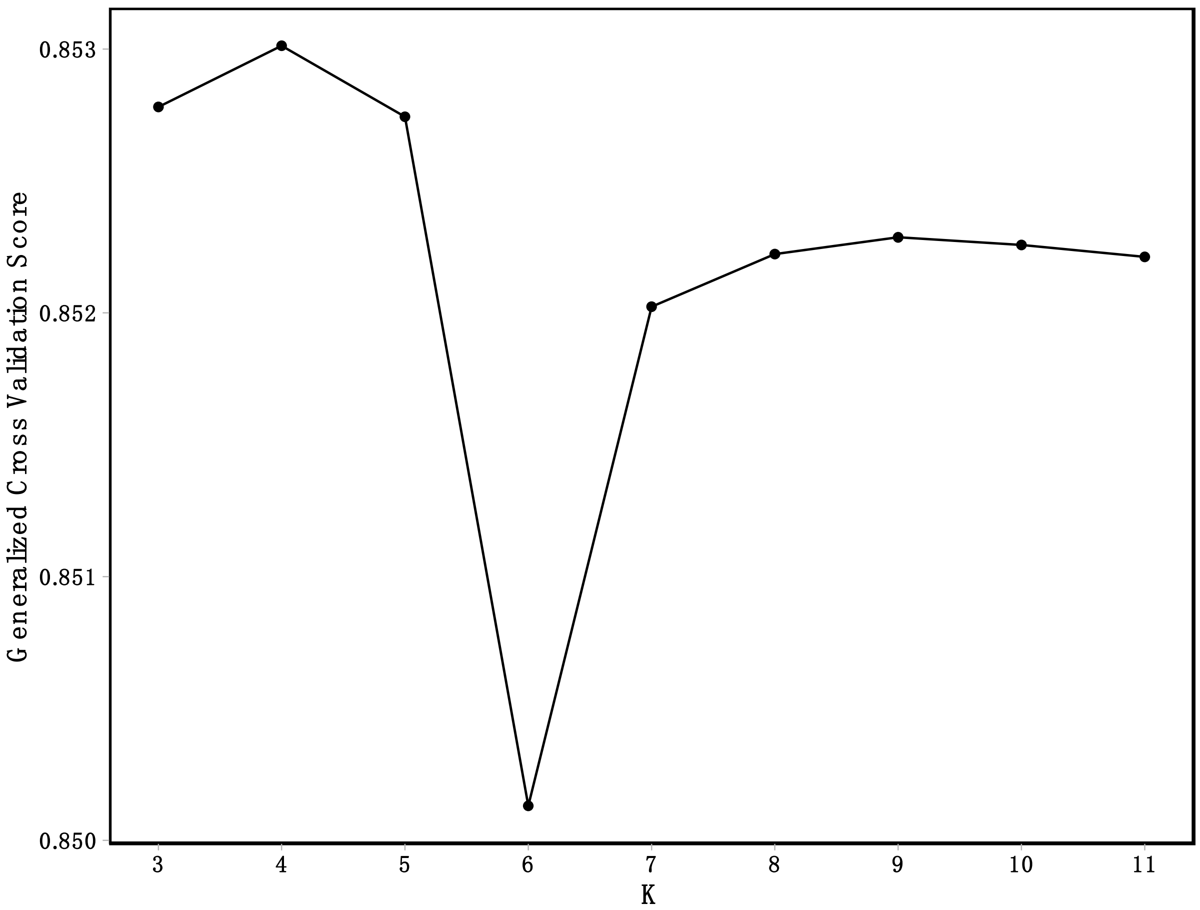

Figure S2. Generalized cross validation scores for different K values (smoothing parameter) of the GAM with rela-tive fitness as a function of standardized sperm count. Lower generalized cross validation score indicates better model fit. The K value with the best fit (6) was used for generating Figure 1.

Table S1. Summary statistics of sperm parameters, body size, and paternity for 171 male *Anolis sagrei*. Means and standard errors of each trait for all males are presented, as well as the mean and standard error of within-male variation (coefficient of variation, CV) calculated for each trait of each male when appropriate.

Trait	Mean ± Standard Error	Within-male variation, mean CV	Range
Sperm Count	$5.97\times10^{6} \pm 2.08\times10^{5}$	NA	$2.00\times10^{4} – 1.59\times10^{7}$
Velocity (VAPi) (μm/sec)	50.33 ± 0.59	0.17 ± 0.0039	25.13 – 71.61
Linearity (%)	45.86 ± 1.01	0.33 ± 0.0068	19.17 – 84.75
Head length (μm)	13.87 ± 0.02	0.017 ± 0.0003	13.09 – 14.43
Midpiece length (μm)	2.49 ± 0.009	0.059 ± 0.001	2.16 – 2.86
Flagellum length (μm)	75.08 ± 0.09	0.011 ± 0.001	70.38 – 77.95
Total sperm length (μm)	91.45 ± 0.09	0.001 ± 0.0001	86.68 – 94.32
Snout-vent length (mm)	55.64 ± 0.36	NA	39 – 65
No. Offspring sired	6.02 ± 0.43	NA	0 – 27

Table S2. Sample sizes for genotyping and phenotyping with exclusion criteria. Potential sires and dams were present between April and October 2015, while 2015 offspring were captured between July-August 2015 and May 2016. Ejaculate sampling occurred only on the May 2015 census trip.

	Included	Excluded	Reason for exclusion
Potential sires captured in 2015	331		
Genotyped	307	24	No DNA (*n* = 19) or >50% missing loci (*n* = 5)
Identified as 2015 sires	200	107	No parentage match in SNPPIT (*n* = 102) or >50 loci (23%) missing (*n* = 5).
Potential dams captured in 2015	638		
Genotyped	580	58	No DNA (*n* = 32) or >50% missing loci (*n* = 26)
Identified as 2015 dams	381	199	No parentage match in SNPPIT (*n* = 185) or >50 loci (23%) missing (*n* = 14).
Offspring from 2015 cohort	2060		
Genotyped	1981	79	No DNA (*n* = 26) or >50% missing loci (*n* = 53)
Assigned parentage at FDR < 0.005	1185	796	No parentage match in SNPPIT
Males captured in May 2015	211		
Sampled for ejaculates	202	9	Immature male or no ejaculate
Phenotyped for sperm count	202	0	
Phenotyped for sperm morphology	198	4	Few or no sperm cells on slide
Phenotyped for sperm velocity	184	18	Sperm crowded or non-motile
Included in selection analysis	171	40	Missing phenotype or genotype
